# Osseous Choristoma of the Tongue: A Review of Etiopathogenesis

**DOI:** 10.1155/2014/373104

**Published:** 2014-12-14

**Authors:** Edoardo Gorini, Mauro Mullace, Luca Migliorini, Emilio Mevio

**Affiliations:** ^1^Department of Otorhinolaryngology, Ospedale Fornaroli, Via Donatore del Sangue, 20013 Magenta, Italy; ^2^Department of Pathology, Ospedale Fornaroli, Via Donatore del Sangue, 20013 Magenta, Italy

## Abstract

Osseous choristoma is a normal bone tissue in an ectopic position. In the oral region lingual localization occurs more frequently and the mass is generally localized on the dorsum of the tongue. Definitive diagnosis is obtained only after histopathologic examination. The etiology remains already debatable. The treatment of choice is surgical excision. In this paper we present a case of tongue osseous choristoma and a review of the literature.

## 1. Introduction

The term choristoma is used to describe the growth of normal tissue in an abnormal position. Choristoma of the mouth may be composed of several different tissue types. These include bone, cartilage, gastric mucosa, glial tissue, and tumor-like masses of sebaceous glands. Osseous choristoma is a well circumscribed benign growth of normal, mature osseous tissue in ectopic sites.

Osseous choristoma of the tongue is an extremely rare condition, of which only 66 cases have been reported in the literature till now. The osseous histotype is the most frequently described among choristomas. In the oral region lingual localization occurs more frequently and the mass is generally localized on the dorsum of the tongue. The etiology remains already debatable. The treatment of choice is surgical excision.

## 2. Case Report

**Figure 1:**
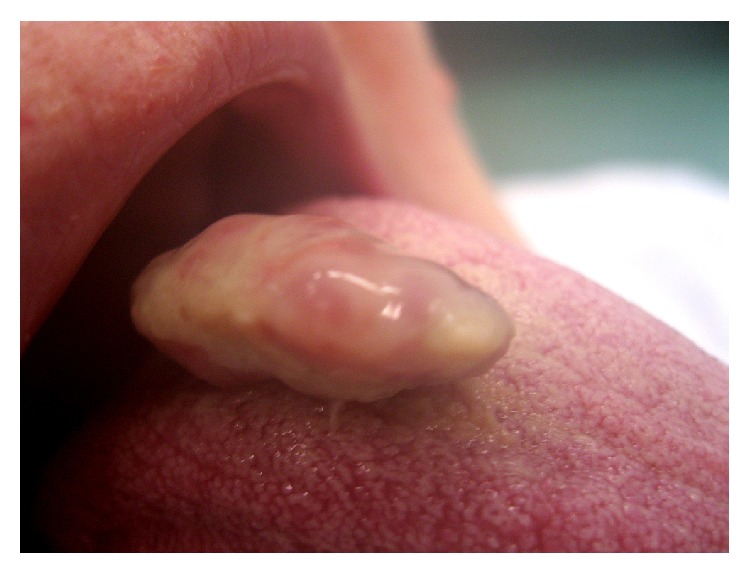
Paramedian dorsum of the tongue choristoma.

**Figure 2:**
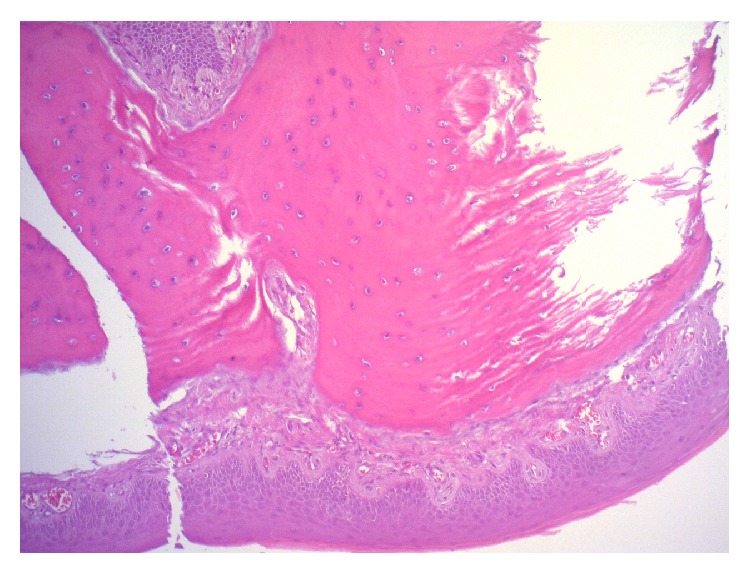
Histological examination shows mature lamella lined by mucous membrane of the tongue.

A 10-year-old girl was referred to our ENT unit for a whitish 1 cm sessile swelling of the paramedian dorsum of the tongue, near foramen caecum (Figure 1). Upon palpation this not ulcerated lesion was firm. The patient did not complain of any symptom for many years. In the last two months she complained of lump. The mother of the girl reported that she noticed the little swelling since first months of life. She underwent neck ultrasound that showed a normal thyroid gland in shape and position. Although she was paucisymptomatic we proposed and performed a surgical excision of the lesion in order to obtain a histological diagnosis. Histological examination described an osseous choristoma of the tongue. The specimen was totally included for histological examination. Histologically, it appeared as a polypoid nodule of cortical bone tissue (mm 10 in maximum diameter) beneath the mucous membrane of the tongue covered by orthokeratinised squamous epithelium. The bone tissue was lamellar type with well developed haversian systems. The bone tissue was with sharply demarcated edges and in the surrounding tissue there was no inflammation or scar tissue (Figure 2).

After 1-year follow-up there is no evidence of recurrence.

## 3. Discussion and Review of the Literature

The term osseous choristoma was introduced by Krolls et al. in 1971 [1]. By definition it is a growth of normal tissue in an abnormal position. Choristoma of the mouth may be composed of several different tissue types. These include bone, cartilage, gastric mucosa, glial tissue, and tumor-like masses of sebaceous glands [2, 3].

Osseous choristoma of the tongue is an extremely rare condition. In the literature 66 cases have been described (Table 1) [1–45]. In our review the patient age ranged from five to seventy-three years (mean age: 28,7 years), with the majority of the patients being in the second or third decades of life. Choristomas of the tongue occur more frequently in women (M:F 16:44).

The most frequent affected region is the posterior third of the tongue dorsum near to the foramen caecum and circumvallate papillae. Pathogenesis of choristoma is still unknown and remains already debatable. Several theories tried to explain the pathogenesis of this disease. Some authors suggested that remnants of the undescended thyroid tissue might produce an osseous lesion but in some rare case choristoma is localized not in midline but on the border of the tongue [46]. In these cases some authors [36, 47] suggested a traumatic pathogenesis. They consider that the posterior third of the tongue is site of traumatic irritation by different lingual movement during swallowing and articulation and that frequent trauma leads to local inflammation with deposit of calcium. This theory cannot explain the formation of osseous choristoma, because this lesion contains fully developed bone with haversian system and not just calcifications.

In our opinion embryologic theory sounds true. Embryologically the tongue is a very complex structure. First and third branchial arches give rise, respectively, to the anterior two-thirds and posterior third of the tongue. It was suggested that pluripotential cells from these arches give origin to the osseous choristoma [5, 12].

Choristoma appears as a sessile or pedunculated mass usually covered by normal mucosa. The sizes of the lesions vary from 3 mm to 50 mm at their largest diameter.

Most of osseous choristoma of the tongue presents as a frequently asymptomatic swelling. The most frequent symptom is lump (46% of cases). Rarely patient complains of dysphagia (5 cases), gagging (4 cases), pain (4 cases), and nausea (1 case). Symptoms are correlated to lesion size, tumour localization, and surrounding tissues flogosis. The differential clinical diagnosis can be also based on the tumor location. When the lesion is located on the dorsal tongue near the foramen caecum we should consider in differential diagnosis benign tumours (hemangioma, lymphangioma, teratoma, hamartoma, and leiomyoma), thyroglossal duct cyst, lingual thyroid, mucocele, pyogenic granuloma, and malignant tumours (rhabdomyosarcoma, other sarcomas, and epidermoid carcinoma) [25]. Traumatic neuroma, neurofibroma, schwannoma, fibroma, and cartilaginous choristoma usually are located on the tongue margin. Pyogenic granuloma, mucocele, and cartilaginous choristoma frequently involve the anterior portion of the tongue. Nevertheless definitive diagnosis is obtained only after histopathologic examination.

The treatment of choice is surgical excision. Recurrence or malignant evolution has not been described.

## Figures and Tables

**Table 1:** 

	Author	Age (y)/sex	Location	Size	Symptom
1	Cataldo et al. [4]	39/F	Posterior tongue	1 cm Ø	None
2	Begel et al. [5]	22/F	Area of CP	1 × 0,5 cm	Dysphagia
3	Jahnke and Daly [6]	22/F	Posterior to CP	1,3 × 0,8 × 0,7 cm	Lump
4	Kaye [7]	26/F	Base of the tongue	1 × 1 cm	Lump
5	Goldberg et al. [8]	65/M	Lateral border	1 cm Ø	None
6	Krolls et al. [1]	22/F	Anterior to CP	0,75 cm Ø	None
7		23/M	Area of FC	Un.	Un.
8		73/M	Posterior tongue	Un.	Gagging
9		9/F	Area of FC	Un.	Gagging
10		25/F	Posterior tongue	0,5 cm Ø	Un.
11		11/F	Posterior tongue	2 cm Ø	Un.
12		23/M	Area of CP	0,5 × 0,5 × 0,5 cm	None
13		39/M	Area of CP	0,6 × 0,6 cm	None
14	Singh and Doyle [9]	14/F	Left border	Un.	Un.
15		22/F	Area of CP	0,5 cm Ø	None
16	McClendon [10]	15/F	Area of FC	1,4 × 0,6 × 0,5 cm	None
17		20/M	Right border	0,7 cm Ø	None
18		46/F	Area of FC	0,6 cm Ø	None
19	Patel and Dane [11]	42/M	Lateral border	1 cm Ø	None
20	Engel and Cherrick [12]	31/M	Mid third right border	2 cm Ø	Lump
21	Busuttil [13]	8/F	Left border	Pea-sized	Lump
22	Ohno et al. [14]	Un.	Dorsum of the root of the tongue	Un.	Un.
23	Sugita et al. [15]	Un.	Un.	Un.	Un.
24	Sato et al. [16]	Un.	Un.	Un.	Un.
25	Esguep et al. [17]	63/F	Right border	0,5 cm Ø	Lump
26	Wasserstein et al. [18]	50/F	Mid third	1,5 × 0,75 cm	Lump
27	Shimono et al. [19]	37/F	Area of FC	1,5 × 1,5 × 0,7 cm	Lump
28	Main [20]	54/F	Posterior to FC	1,5 cm Ø	Lump
29	Sheridan [21]	20/F	Anterior to CP	1 cm Ø	Lump
30	Cabbabe et al. [22]	5/F	Base of tongue	0,6 × 0,5 × 0,3 cm	Lump
31	Nash et al. [23]	31/M	Right border	2,5 cm Ø	None
32	Weitzner [24]	52/F	Mid third	Small nodule	None
33		25/F	Posterior tongue	0,8 × 0,4 × 0,4 cm	Lump
34		27/F	Posterior tongue	0,8 × 0,7 × 0,3 cm	Lump
35	Tohill et al. [25]	31/F	Anterior to CP	1 × 0,8 × 0,7 cm	None
36	Markaki et al. [26]	25/F	Posterior to CP	0,8 × 0,4 × 0,3 cm	Lump
37	Van Der Wal and van der Waal [27]	31/F	Area of FC	1 cm Ø	Lump
38	Cannon and Niparko [28]	51/F	Posterior tongue	Un.	Lump
39	Bernard et al. [29]	21/F	Area of FC	2 cm Ø	Lump
40	Maqbool et al. [30]	8/F	Right vallecula	5 × 4 cm	Dysphagia, distress
41	Lutcavage and Fulbright [31]	11/F	Posterior to FC	1 cm Ø	Lump
42	Ishikawa et al. [32]	53/F	Area of FC	0,8 cm Ø	Foreign body sensation
43		5/F	Anterior to CP	3 mm Ø	Lump
44	Lee et al. [33]	35/M	Lateral border	Un.	Lump
45	Ngeow et al. [34]	Un.	Un.	Un.	Un.
46	Manganaro [35]	Un.	Un.	Un.	Un.
47	Vered et al. [36]	44/M	Left border	0,7 × 0,7 × 0,6 cm	Gagging, nausea, and dysphagia
48		27/M	Posterior to CP	1 × 0,5 cm	Pain, gagging
49	Supiyaphun et al. [3]	28/F	Area of FC	1 × 0,8 × 0,6 cm	Throat irritation
50		25/F	Area of FC	0,7 × 0,5 × 0,4 cm	Lump
51		9/F	Area of FC	0,7 × 0,6 × 0,5 cm	None
52		35/F	Area of FC	0,7 × 0,5 × 0,5 cm	None
53		27/F	Area of FC	1,2 × 0,9 × 0,6 cm	None
54		21/F	Area of FC	1,5 × 1,3 × 0,8 cm	Lump
55		22/M	Area of FC	0,9 × 0,8 × 0,6 cm	None
56		19/F	Area of FC	1,1 × 0,7 × 0,7 cm	None
57	Lin et al. [37]	Un.	Posterior tongue	Un.	Un.
58	Horn et al. [38]	11/F	Posterior tongue	Un.	Lump
59	Benamer and Elmangoush [39]	14/F	Mid third	1 cm Ø	Lump
60	Carvalho et al. [40]	22/F	Posterior tongue	1 cm Ø	None
61	Andressakis et al. [2]	72/M	Anterior to CP	1,5 × 1 cm	Pain dysphagia
62	Naik et al. [41]	25/F	Posterior tongue	1,2 × 1,1 × 0,5 cm	Lump
63	Chen et al. [42]	57/F	Posterior tongue	1 cm Ø	Lump, dysphagia, and odynophagia
64	Spencer and Reed [43]	11/M	Posterior tongue	1,1 × 0,9 × 0,6 cm	None
65	Lin et al. [44]	15/M	Area of FC	0,5 × 0,5 cm	Lump
66	Qin et al. [45]	Un.	Un.	Un.	Un.
67	Present case	10/F	Anterior to FC	1 cm Ø	Lump

(CP: circumvallate papillae, FC: foramen caecum, and Un.: unknown).
